# Promoting the Development of *Astragalus mongholicus* Bunge Industry in Guyang County (China) Based on MaxEnt and Remote Sensing

**DOI:** 10.3389/fpls.2022.908114

**Published:** 2022-07-07

**Authors:** Ru Zhang, Mingxu Zhang, Yumei Yan, Yuan Chen, Linlin Jiang, Xinxin Wei, Xiaobo Zhang, Huanting Li, Minhui Li

**Affiliations:** ^1^Baotou Medical College, Baotou, China; ^2^Inner Mongolia Hospital of Traditional Chinese Medicine, Hohhot, China; ^3^Inner Mongolia Key Laboratory of Characteristic Geoherbs Resources Protection and Utilization, Baotou, China; ^4^Department of Pharmacy, Inner Mongolia Medical University, Hohhot, China; ^5^School of Life Sciences, Inner Mongolia University, Hohhot, China; ^6^State Key Laboratory Breeding Base of Dao-di Herbs, National Resource Center for Chinese Materia Medica, China Academy of Chinese Medical Sciences, Beijing, China

**Keywords:** *Astragalus mongholicus* Bunge, value chains, species distribution models, maximum entropy, remote sensing, random forest

## Abstract

To provide high-quality *Astragalus mongholicus* Bunge to domestic and foreign markets and maintain sustainable development of the *A. mongholicus* industry, Firstly, we evaluated the impact of environmental factors and planting areas on the *A. mongholicus* industry. The maximum entropy method (MaxEnt) was utilized to simulate the suitability distribution of *A. mongholicus* and establish the relationship between the active component contents of *A. mongholicus* and ecological factors through linear regression analysis. The random forest algorithm was subsequently used to perform feature selection and classification extraction on Sentinel-2 imagery covering the study area. Furthermore, the planting, processing, and sales of *A. mongholicus* in Guyang County were investigated, and the roles of stakeholders in the value chains were analyzed. The results demonstrated that precipitation of the warmest quarter, minimum temperature of the coldest month, standard deviation of seasonal temperature changes, range of mean annual temperature, and mean diurnal range [mean of monthly (max temp - min temp)] were the five environmental variables that contributed the most to the growth of *A. mongholicus*. The most influential factor on the distribution of high-quality *A. mongholicus* was the mean temperature of the coldest quarter. The classification results of image features showed that the planting areas of *A. mongholicus* was consistent with the suitable planting areas predicted by MaxEnt, which can provide data support to the relevant departments for the macro development of the *A. mongholicus* industry. In the production of *A. mongholicus*, 10 value chains were constructed, and the study demonstrated that the behavior of stakeholders, target markets, and the selected planting area had a significant impact on the quality of *A. mongholicus*.

## Introduction

**Figure 1:**
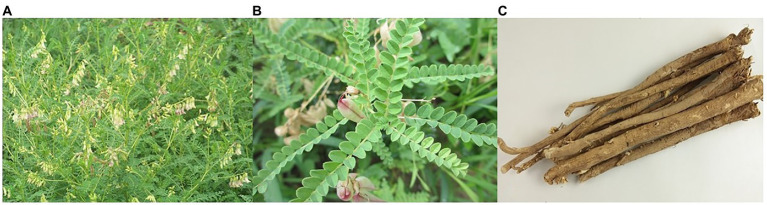
The cultivated of *Astragalus mongholicus* **(A)**; Details of *A. mongholicus* **(B)**; dried roots *A. mongholicus* **(C)**.

The continuous improvement of people’s health awareness has resulted in medicinal plants receiving increased attention. *A. mongholicus* is an important raw material for use in functional health care products and is widely sought after. In China, the root of *A. mongholicus* is a traditional Chinese medicine (Figure 1). Moreover, *A. mongholicus* plants are rich in glycosides, polysaccharides, and flavonoids that exert various beneficial health effects, such as immunoregulation, protection against cardiovascular and cerebrovascular diseases, delayed aging, and anti-rheumatism effects (Durazzo et al., 2021).

In recent years, the growth of international and domestic market demand for *A. mongholicus* has prompted the expansion of its plantation areas, in an effort to alleviate the supply and demand pressures. Guyang County is renowned as one of the main producing areas of high-quality *A. mongholicus*. Accordingly, the *A. mongholicus* produced in Guyang County was awarded the “Geographical Indication of Agricultural Products” status by the Ministry of Agriculture and Rural Affairs in 2017.^1^ Furthermore, subsequent to its review as a high-quality agricultural product by the working institutions of the prefecture- and provincial-level agricultural and rural departments in September 2020, *A. mongholicus* from Guyang County was included in the second batch of national famous, special, and excellent new agricultural products.^2^ Therefore, Guyang county is considered an ideal location for the production of high-quality *A. mongholicus* (Dao-di Herbs).

Due to the rotation cultivation mode of *A. mongholicus*, a large number of suitable land is needed to provide options for planting areas in the coming years. However, to obtain the planting situation of *A. mongholicus*, it also needs sufficient time to consult the data and conduct field investigation. This survey method is expensive, inefficient, and prone to subjective deviation, resulting in errors in planting conditions. Species distribution models (SDMs) use specific algorithms to correlate species distribution with environmental variables, thereby predicting the potential distribution of a species across different geographical spaces and time (Zhu et al., 2013). This probability reaction reflects habitat suitability and allows for further research on the regional distribution of the estimated model of the target species. Among the SDMs, the maximum entropy model (MaxEnt) is most commonly used (Xu et al., 2015) which produces good results. For example, Yang et al. (2020b) successfully used the MaxEnt model to predict the habitat suitability of *Salweenia bouffordiana* by analyzing the main environmental factors affecting its habitat (Yang et al., 2020b). Considering this, using the MaxEnt model to understand the relationship between different environmental factors and species distribution is an effective way to develop management and protection strategies for medicinal plant species.

Remote sensing can play significant roles in determining the distribution, growth area and status, and occurrence of diseases and insect pests among medicinal plants. Since 2000, remote sensing technology has gradually advanced the theory and methods of medicinal plant resource investigations and has been effectively applied in medicinal plant production (Lan et al., 2021). Previous studies have successfully utilized Landsat-8TM, Gaofen-1 (GF-1), Resources satellite three (ZY-3), and other remote sensing satellite data for the detection of medicinal plants (Na et al., 2013; Shi et al., 2017). Sentinel-2 is mainly used for land environmental monitoring and provides information on land cover, including soil condition and vegetation patterns. This information is crucial for improving agricultural and forestry planting structures, estimating agricultural areas, and predicting crop yield. On this basis, using Sentinel-2 imagery to comprehensively monitor the planting distribution of *A. mongholicus* in Guyang County may be of great significance to the development of the *A. mongholicus* industry.

With the exception of planting area, planting personnel, processing, sales and other factors also affect the development of *A. mongholicus* industry. Currently, *A. mongholicus* is mainly cultivated by individual farmers, and only a few large-scale cultivation enterprises exist. These individual farmers are restricted by limited funds, small-scale planting, and lack of cultivation knowledge, and the consequential blind planting results in failure to meet standardized planting requirements. In addition, variety mixing and species degradation in the seed supply base (Qi, 2020), the use of inappropriate planting habitats, and dense application of herbicides, fertilizers, and insecticides may reduce crop quality and be detrimental to consumer health (Zhao et al., 2017). In contrast, large-scale planting companies have mainly been able to circumvent these problems by hiring experienced professionals and utilizing specialized mechanical equipment to meet planting standards. Medicinal plant products pass through several stakeholder levels, including processing and distribution, before finally reaching the consumer. The processing of *A. mongholicus* is primarily not very extensive, and is mainly executed in family workshops using relatively outdated technology. Moreover, the foundational processing technologies of some enterprises also requires improvement to avoid possible imbalances in the quality of medicinal materials (Sun and Chen, 2018). The value chain (VC) describes the entire process from the initial planting of raw materials to the processing and sale of final products, all while considering the relationships between different stakeholders in the chain (Booker et al., 2012). In recent years, an increasing number of people are applying the VCs to the Chinese herbal medicine industry. According to the production and circulation mode, the roles of different stakeholders in the VCs are organized to effectively evaluate the quality and economic benefits of Chinese herbal medicine in the different VCs (Booker et al., 2015).

In addition to the utilization of *A. mongholicus* as a traditional Chinese medicine, it has been incorporated into Chinese patent medicine, food therapeutics, and other health care products (Qin et al., 2013), resulting in a gradual expansion of its market demand. The artificial planting of high-quality *A. mongholicus* may be an effective method to bridge the gap between supply and demand. To this end, this study aims to (1) predict the suitable growth area of *A. mongholicus* by utilizing MaxEnt and monitoring ecological factors affecting its production. Linear regression analysis was used to evaluate the relationship between the active component content of *A. mongholicus* and ecological factors, and to identify those ecological factors that contributed most to the growth of *A. mongholicus*; (2) monitor planting distribution areas by combining Sentinel-2 imagery; and (3) visit and inspect the planting, processing, and sales links of *A. mongholicus*, build VCs, and analyze the impact of stakeholder behavior on the development of the *A. mongholicus* industry.

## Materials and Methods

### Study Area Description

**Figure 2:**
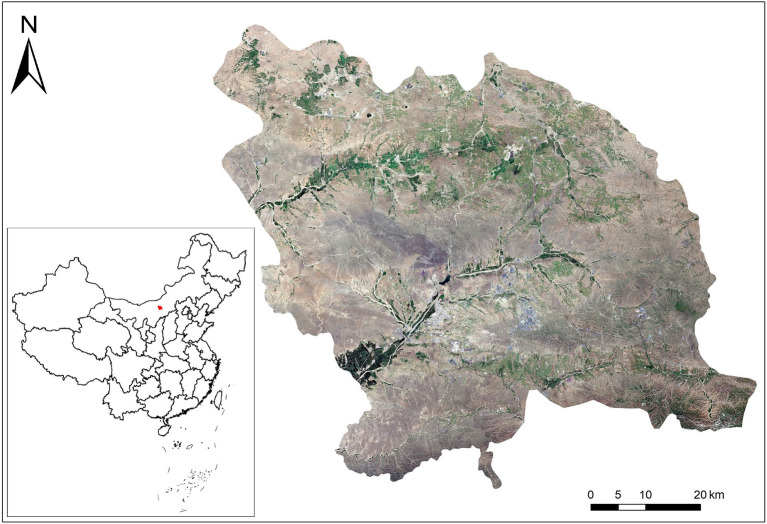
Land cover map of Guyang County.

Guyang County is located in central Inner Mongolia (Figure 2), at latitude 40°42′–41°08′ N and longitude 109°40′–110°41′ E. The county stretches across approximately 80 km (east to west) and is roughly 66 km wide (north to south), covering a total area of 5,025 km^2^. Guyang county has a mid-temperate, continental, arid, and semi-arid monsoon climate, with low average temperatures, little precipitation, and sufficient sunlight. Furthermore, major temperature variations are characteristic of Guyang county, and its mountains and hills make up approximately 90% of the total area (Rural Social and Economic Investigation Department of National Bureau of Statistics, 2021). Chunkun Mountain, in the east of the territory, is 2,321 m above sea level, dividing the county into the southern mountainous and northern hilly areas. The area in between constitutes the Guyang and Bailingnuo basins, of which the lowest point is 1,240 m above sea level. Guyang presents with a typical “plateau basin” terrain that performs an adequate heat collection function, which is beneficial to the growth of *A. mongholicus*.

### Study Species and Data Collection

In this study, the sample data were derived from a field survey of Guyang county from July 8 to July 12, 2021 using the Global Positioning System (GPS), from which the geographic distribution data of *A. mongholicus* were obtained. During the positioning process, sample points were randomly selected from each plot subject to the condition that the minimum distance between any two points would be at least 20 m. Subsequently, from 8 October to 12 October 2021, samples of mature *A. mongholicus* from Guyang County were collected for active ingredient content determination. Different stakeholders of the *A. mongholicus* industry in Guyang County were interviewed, and relevant information on the cultivation, processing, and sales stages of *A. mongholicus* was obtained using field investigations.

### MaxEnt Predicted *Astragalus mongholicus* Distribution

Climate is the main environmental factor determining species distribution. The MaxEnt model was used to explore the environmental niche and potential distribution of *A. mongholicus* according to its spatial location and environmental variables, thereby allowing for the selection of a suitable planting area for *A. mongholicus*.

#### Ecological Factor Selection

A total of 19 climate (with a resolution of 30 s) and altitude datasets were obtained from the global climate data website.^3^ We selected 20 ecological factors, including temperature, precipitation, and altitude, to determine which ecological factors have the greatest impact on the development of the planting industry (Ngarega et al., 2021; Zhang et al., 2021).

#### MaxEnt Model Prediction

Jaynes et al. proposed the MaxEnt principle in 1957 (Jaynes, 1957), and Phillips et al. developed MaxEnt software—that is easy to operate and does calculations swiftly—using the Java^™^ programming language (Phillips et al., 2006), based on the original model. Herein, MaxEnt software (Version 3.4.1 K) was used to predict the suitable distribution of *A. mongholicus*, and 10-fold cross-validation was conducted to analyze the validity and accuracy of the model. The jackknife method was used to measure the weight of each variable and output it in logistic format. The maximum number of iterations and the convergence domain were set to 10^5^ and 0.0005, respectively. The operation was repeated 10 times, and the remaining values were used as default values to extract and analyze habitat suitability. Using the latitudinal and longitudinal geographical distribution data of *A. mongholicus* and relevant ecological factor data from the study area, a prediction model was established for the potential geographical distribution of *A. mongholicus* (Zhu et al., 2017). The model results were evaluated using the area under the receiver operating characteristic (ROC) curve (area under curve, AUC). The AUC-based evaluation standard indicated whether the prediction result was less accurate (<0.5), acceptable (0.5–0.8), or ideal (0.8–0.9), the latter of which would demonstrate high modeling accuracy.

#### Construction of Active Component Content and Its Relationship With Main Ecological Factors

Saponins and flavonoids are the main active components in *A. mongholicus*, which serve as valuable indices for evaluating the quality of *A. mongholicus* in Chinese, British, and European pharmacopoeia. According to the “Chinese Pharmacopoeia” (2020 edition), the astragaloside IV and calycosin-7-glucoside content in 37 *A. mongholicus* samples had been determined using high-performance liquid chromatography (HPLC; European Pharmacopoeia Commission, 2017; British Pharmacopoeia Commission, 2019; National Pharmacopoeia Committee, 2020). In the present study, astragaloside IV was determined using the Thermo Fisher Ultimate 3,000 HPLC system, Agilent C18 column, with a flow rate of 1.0 ml/min. The mobile phase consisted of acetonitrile and deionized water (34%:66%), and the injection volume was 10 μl. Furthermore, calycosin-7-glucoside was separated on a Waters C18 column at a flow rate of 1 ml/min. The mobile phase consisted of acetonitrile (solvent A) and water containing 0.2% methanoic acid (solvent B). Gradient elution was applied as follows: 0–20 min, 80–60% B; 20–30 min, 60% B. The injection volume was 10 μl and the temperature of the column was maintained at 30°C. Detection was performed at a wavelength of 260 nm and each sample was assayed in triplicate.

In addition, SPSS statistical analysis software was utilized to analyze the differences in astragaloside IV and calycosin-7-glucoside contents in *A. mongholicus* from different township areas in Guyang County. A correlation matrix was used to determine the relationship between astragaloside IV, calycosin-7-glucoside, and the major ecological factors affecting *A. mongholicus* growth. By conducting stepwise linear regression analysis, the relationship equations between each index component and the main ecological factors were obtained.

The relationship equations were input into the grid calculator of ArcGIS to obtain the quantitative distribution layers of astragaloside IV and calycoside-7-glucoside in *A. mongholicus*. Using the spatial calculation function of ArcGIS, these two layers were superimposed on the ecological suitability distribution layer of *A. mongholicus*, from which the spatial suitability distribution region of astragaloside IV and calycoside-7-glucoside in Guyang County was obtained.

### Acquisition and Processing of Remote Sensing Data

#### Image Data Acquisition

In recent years, the continuous development of remote sensing technology and its applications has greatly reduced the human error of traditional manual field surveillance, improved the objectivity, scientificity, and accuracy of survey data, and has been widely used to survey medicinal plant resources (Chen, 2021). We aimed to use remote sensing technology to monitor the planting area of *A. mongholicus*, obtain data on its planting practices and area, provide references for relevant departments to formulate related policies and plans, ensure product supply and stable market prices, and promote the steady development of the *A. mongholicus* industry.

The Sentinel-2 satellite was launched by the European Space Agency (ESA) for the EU Copernicus Programme to support global land services, including the monitoring of vegetation, soil and water coverage, inland waterways, and coastal areas. Sentinel-2 imagery cover 13 spectral bands of visible light. Moreover, considering the good spatial resolution, global coverage, and relatively good time resolution of Sentinel-2 data, it is widely used in many fields.

Images covering the entire Guyang County (February–November 2021) were downloaded from the Copernicus Open Access Center.^4^ Although some associated data have been orthogonal and geometric corrected, they cannot be directly used. As such, preprocessing, such as atmospheric correction and resampling, was required and the resolution of all bands was resampled to 10 meters. The pre-processed image was synthesized by the layer stacking function of ENVI. Additionally, bands 2, 3, 4, 5, 6, 7, 8, 8A, 11, and 12 were mainly included, whereafter regional cropping was performed using the subset data of the ROIs function to obtain a complete image of Guyang County.

#### Time Window Selection for Extraction of the Vegetation Index

According to phenological knowledge, the appropriate time window is selected to extract the characteristics of crop planting structure during the process of crop development. To ensure economic benefits, crop phenological indicators were recorded through field observations. Most *A. mongholicus* in Guyang County were selected and sown in April–May, matured in July–August, and harvested in October–November. Various crops, in addition to *A. mongholicus*, are planted in Guyang County. To allow for better classifications, we extracted the normalized difference vegetation index (NDVI) of Guyang County from February to October 2021 using ENVI software and combined the training datasets of *A. mongholicus* to develop the temporal NDVI profile, thereby obtaining the spectral changes and time windows over different periods (Inoue and Olioso, 2006). The NDVI is used to detect the growth state of vegetation and vegetation coverage. Moreover, it can reflect the background effects of the vegetation canopy, such as soil, wetland, snow, dead leaves, and roughness, etc. In addition, we also extracted the ratio vegetation index (RVI), enhanced vegetation index (EVI), and normalized red edge vegetation index (NDVIre; Table 1; Tan et al., 2018).

**Table 1:** Spectral characteristic index set.

Spectral region	Vegetation index	Formula
Normalized vegetation index	NDVI	(B8-B4)/(B8 + B4)
Enhanced vegetation index	EVI	2.5 × (B8-B4)/(B2 + 6 × B1 + 7.5 + B3 + 1)
Ratio vegetation index	RVI	B8/B4
Red edge vegetation index 1	NDVIre1	(B8 − B5)/(B8 + B5)
Normalized difference vegetation index red-edge 1narrow	NDVIre 1n	(B8A − B5)/(B8A + B5)
Normalized difference vegetation index red-edge 2	NDVIre 2	(B8 − B6)/(B8 + B6)
Normalized difference vegetation index red-edge 2narrow	NDVIre 2n	(B8A − B6)/(B8A + B6)
Normalized difference vegetation index red-edge 3	NDVIre 3	(B8 − B7)/(B8 + B7)
Normalized difference vegetation index red-edge 3narrow	NDVIre 3n	(B8A − B7)/(B8A + B7)

#### Principal Component Analysis

Principal component analysis (PCA) was performed on the Sentinel-2 imagery within the optimal time window. PCA is a commonly used dimensionality reduction method in image processing. In remote sensing image classification, PCA is often used to eliminate the correlation between bands and perform feature selection. Under the premise of not reducing the “effective” information, the original dataset was converted into “effective” information, and the identification was carried out with fewer dimensions, thereby reducing the number and dimension (Hess and Hess, 2018; Wang et al., 2021a).

#### Texture Feature Extraction

Texture features were extracted from the bands containing the main features, based on the PCA results. Texture reflects a certain change rule for an object’s surface color and gray level. This information effectively distinguishes ground objects with similar spectra and different spatial distribution structure characteristics, and is widely used to extract image information. Texture features are also important in the process of feature extraction, representing the spatial distribution of pixels in remote sensing images. The addition of texture features can aid in the reduction of the salt and pepper noise and improve classification accuracy (Liu et al., 2021).

### Classifier Classification

The random forest classifier (Breiman, 2001), which has the important function of analyzing the significance of features and building a classification regression tree, was used. The collected coordinate points were processed using ArcGIS to generate a sample set, and 70% of the sample points were randomly selected and imported into EnMAP and ENVI software together with remote sensing images as training sets.

### Accuracy Evaluation

A confusion matrix was used to classify and identify the planting area of *A. mongholicus*. The matrix consisted of the following terms: overall accuracy (OA), user accuracy (UA), and the Kappa coefficient of variation (Kappa; Vasileios et al., 2018). The overall accuracy represents the probability that the classified result is consistent with the test data type for each random sample. Furthermore, Kappa coefficient is an indicator for comprehensively evaluating the classification accuracy and is used to judge the consistency of images.

### VCs Analysis

First, we determined the main links in the production process of *A. mongholicus*, whereafter the stakeholders in the process were identified and matched to different chains, linking the main production activities with the stakeholders according to their different interests. A VC analysis diagram of the roles played by the stakeholders in different chains was then created. In addition, the production behavior, quality, and financial performance of *A. mongholicus* in the VCs were analyzed according to the methods described by Yao et al. (2018) Finally, we analyzed the strengths and weaknesses of the VCs in terms of safety, quality, and geographical indications (Yao et al., 2018).

### Price Forecasting

Fluctuations in the price of Chinese herbal medicines not only have a major impact on stakeholders in the VCs, but also exert pressure on governmental market regulation, affecting the sustainability and healthy development of the Chinese medicine industry (Cui et al., 2020). Therefore, the price forecast of Chinese herbal medicines could provide a price reference for stakeholders in the VCs and ensure smooth progression of Chinese herbal medicines planting and production processes. The autoregressive integrated moving average model (ARIMA) is a time-series autoregressive technique that calculates future short-term forecasts by analyzing historical data (Alabdulrazzaq et al., 2021). It was created by Box and Jenkins in the 1970s to mathematically describe changes in a time series (Rao et al., 1972). In the current study, price data of *A. mongholicus* from 2017 to 2021 was collected and the future market price for *A. mongholicus* was predicted and analyzed using the ARIMA model in SPSS (IBM, Armonk, NY, United States). It is worth noting that we first used the price data from 2017–2020 to make a price forecast for 2021, and compared the forecast results with the price data we collected to verify the accuracy of the model. Then, we forecast the price of *A. mongholicus* in 2022 based on the data from 2017–2021.

## Results

### Study on Ecological Suitability of *Astragalus mongholicus*

The MaxEnt model prediction results were imported into ArcGIS software (George and Fred, 1971). To guide local governments and farmers toward more effective *A. mongholicus* planting strategies in the current environment, and develop medicinal value according to the regional results of the ecological suitability of *A. mongholicus*, Guyang County administrative data were covered in ArcGIS. In calculating the model results, the influence of each ecological factor on the distribution of *A. mongholicus* was determined by analyzing the response curves of those ecological factors that had a notable contribution rate and were ranked high in importance.

#### Accuracy of MaxEnt Model

**Figure 3:**
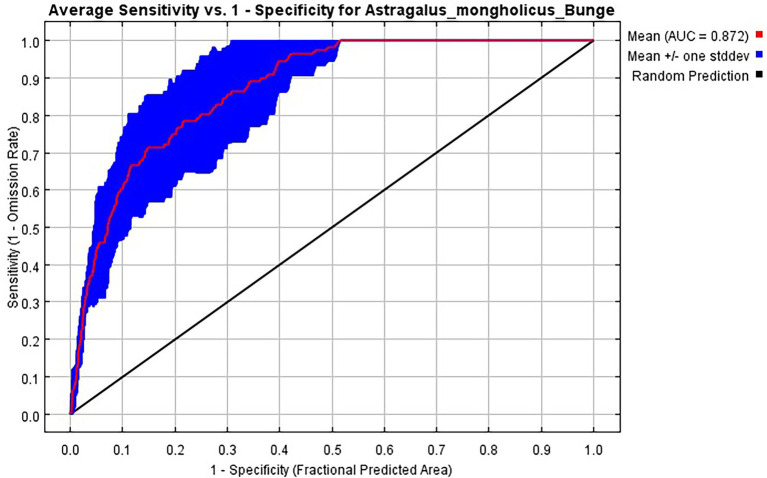
ROC value of *A. mongholicus* modeled by MaxEnt based on distribution date.

The ROC curve showed that the area under the curve (AUC) value of the *A. mongholicus* test sample was 0.872. According to the AUC evaluation standard, the MaxEnt model results were ideal and reached a good level, which also demonstrated the validity of the model for evaluating the habitat suitability of *A. mongholicus* (Figure 3).

#### Regional Ecological Suitability for *Astragalus mongholicus* in Guyang County

**Figure 4:**
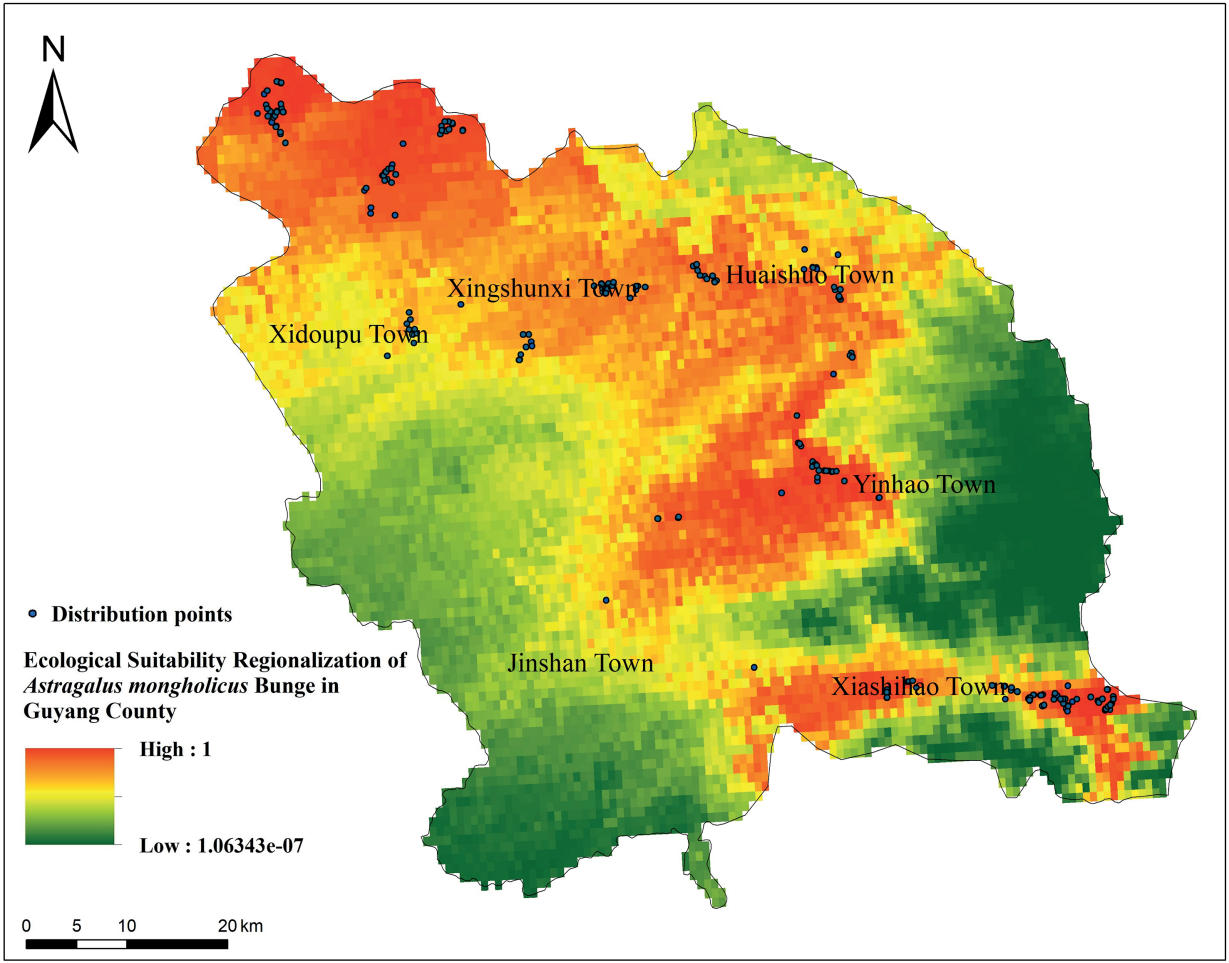
Ecological Suitability Regionalization of *A. mongholicus* in Guyang County.

The model analysis results demonstrated that the north of Xidoupu Town, the west of Yinhao Town, and the middle of Xiashihao Town had higher ecological adaptability. Xingshunxi Town and Huaishuo Town had a large area of medium growth area suitability (Figure 4).

#### Main Ecological Factors Affecting the Growth of *Astragalus mongholicus*

Precipitation of the warmest quarter (26.6%), minimum temperature of the coldest month (20.3%), standard deviation of seasonal temperature changes (11%), range of mean annual temperature (10.8%), and mean diurnal range [mean of monthly (max temp - min temp)] (7.4%) were identified as the five environmental variables with the highest contribution rates to the MaxEnt modelling results. Moreover, their cumulative contribution was 76.1%. These results indicated that the identified environmental variables were the main ecological factors affecting the habitat of *A. mongholicus* (Table 2).

**Table 2:** Details of the 20 ecological factors used to predict *Astragalus mongholicus* distribution.

Abbreviation	Name	Relative contribution	Type
BIO1	Mean annual temperature	0.8%	Continuous
BIO2	Mean Diurnal Range (Mean of monthly (max temp - min temp))	7.4%	Continuous
BIO3	Isothermality	0.5%	Continuous
BIO4	Standard deviation of seasonal changes in temperature	11%	Continuous
BIO5	Maximum temperature of the warmest month	0.6%	Continuous
BIO6	Minimum temperature of the coldest month	20.3%	Continuous
BIO7	Range of mean annual temperature	10.8%	Continuous
BIO8	Mean temperature of the wettest quarter	5.7%	Continuous
BIO9	Mean temperature of the driest quarter	0.9%	Continuous
BIO10	Mean temperature of the warmest quarter	0.4%	Continuous
BIO11	Mean temperature of the coldest quarter	0%	Continuous
BIO12	Mean annual precipitation	0.2%	Continuous
BIO13	Precipitation of the wettest month	2.3%	Continuous
BIO14	Precipitation of the driest month	6.1%	Continuous
BIO15	Precipitation Seasonality (Coefficient of Variation)	1.2%	Continuous
BIO16	Precipitation of the wettest quarter	1.2%	Continuous
BIO17	Precipitation of the driest quarter	0.3%	Continuous
BIO18	Precipitation of the warmest quarter	26.6%	Continuous
BIO19	Precipitation of the coldest quarter	3.2%	Continuous
BIO20	Altitude	0.5%	Continuous

#### Content of Index Components and Relationships With Main Ecological Factors

The astragaloside IV and calycaryin-7-glucoside contents of the 37 *A. mongholicus* samples that were evaluated, are shown in the Supplementary Table 1. The relationships between the astragaloside IV and isoflavone glycoside contents of *A. mongholicus*, respectively, and the main ecological factors are represented by the following equations:


$$\begin{array}{l} y_{1}=\mathrm{0.024}x_{1-}\mathrm{0.016}x_{2}+\mathrm{0.012}x_{3}+\mathrm{0.009}x_{4}\\ \;\;\;\;\;\;+\mathrm{0.007}x_{5-}\mathrm{0.009}x_{6-}\mathrm{0.029}x_{7-}\mathrm{0.003}x_{8-}\mathrm{0.003}x_{9}\\ \;\;\;\;\;\;+\mathrm{0.012}x_{\mathrm{10}}+\mathrm{0.013}x_{\mathrm{11}-}\mathrm{0.001}x_{\mathrm{12}-}\mathrm{0.363}\\ \left(\begin{array}{l} R^{2}=\mathrm{0.491},p\leq \mathrm{0.05};y_{1}:calycaryin-7-glucoside;x_{1}:\\ \;\;\;\;\;\;\;\;\;\;\;\;Bio1;x_{2}:Bio2;x_{3}:Bio3;x_{4}:Bio6;x_{5}:Bio7;x_{6}:\\ \;\;\;\;\;\;\;\;\;\;\;\;Bio\mathrm{10};x_{7}:Bio\mathrm{11};x_{8}:Bio\mathrm{13};x_{9}:Bio\mathrm{14};x_{\mathrm{10}}:\\ \;\;\;\;\;\;\;\;\;\;\;\;Bio\mathrm{16};x_{\mathrm{11}}:Bio\mathrm{18};x_{\mathrm{12}}:Bio\mathrm{19} \end{array}\right). \end{array}$$

$$\begin{array}{l} y_{2}=-0.068x_{1}+0.047x_{2-}0.005x_{3}\\ \;\;\;+0.001x_{4}+0.038x_{5-}0.004x_{6}\\ \;\;\;+0.001x_{7-}0.002x_{8-}0.006x_{9}\\ \;\;\;+0.021x_{10-}0.017x_{11-}0.025x_{12-}0.035x_{13}\\ \;\;\;+0.009x_{14}+0.026x_{15}+0.013x_{16}\\ \;\;\;+0.049x_{17-}0.001x_{20}+4.224\\ \;\;\;\;\;\;\left(\begin{array}{l} R^{2}=0.379,p\leq 0.05;y_{2}:\\ astragalosideIV;x_{2}:Bio1;x_{2}:\\ Bio2;x_{3}:Bio3;x_{4}:Bio4;x_{5}:\\ Bio6;x_{6}:Bio7;x_{7}:Bio8;x_{8}:\\ Bio9;x_{9}:Bio10;x_{10}:Bio11;x_{11}:\\ Bio12;x_{12}:Bio13;x_{13}:Bio14;x_{14}:\\ Bio15;x_{15}:Bio16;x_{16}:Bio18;x_{17}:\\ Bio19;x_{20}:altitude \end{array}\right). \end{array}$$

**Figure 5:**
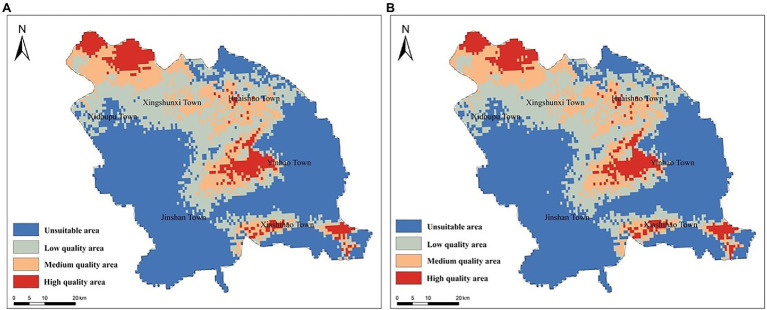
Regional suitability distribution of high-quality *A. mongholicus* in Guyang County. Its suitability was based on the content of astragaloside IV **(A)** and calycosin-7-glucoside **(B)**.

These equations indicate that most ecological factors exerted different effects on the accumulation of the two active substances. For example, the mean temperature of the coldest quarter (BIO11) played an important role in the accumulation of both active compounds; it was negatively correlated with the accumulation of astragaloside IV and positively correlated with that of isoflavone glycoside. Mean annual temperature (BIO1) had a greater impact on the astragaloside IV content, to which it was negatively correlated. Mean diurnal range [mean of monthly (max temp - min temp)] (BIO2), precipitation of the coldest quarter (BIO19), and minimum temperature of the coldest month (BIO6) had positive effects on the accumulation of astragaloside IV. Moreover, mean annual temperature (BIO1) was positively correlated with the accumulation of calycosin-7-glucoside. As indicated in Figure 5, the most suitable areas for the accumulation of the two compounds are similar. The areas with high compound content are mainly distributed in the north and central areas of Guyang County, while the southern area is relatively small. Therefore, cultivation of *A. mongholicus* with high-quality active compounds is mainly suitable in the north of Xidoupu Town, the west of Yinhao Town, and the middle of Xiashihao Town.

### Image Feature Analysis

Remote sensing technology can obtain the dynamic change information of the *A. mongholicus* planting area over time, and further provide basic data for the layout formulation of the *A. mongholicus* planting industry, and the establishment of a high-quality *A. mongholicus* cultivation technology system. Based on the spectral analysis results, months with large spectral differences (June, July, August, September, and October) were further analyzed. The images were collected on June 17, July 30, August 21, September 30, and October 20, 2021. First, PCA was performed on the original images associated with the selected 5 months to obtain those bands with a larger monthly contribution rate, whereafter the texture features of these bands were analyzed. The raw images, vegetation index bands, and texture feature bands for the selected 5 months were then fused.

#### Principal Component Analysis

PCA was used in this study because it both aids selection of useful features and improves separability in the transformed feature space. In addition, PCA is beneficial in terms of its operation, because it is an unsupervised analysis. This method generates super pixels by simple linear iterative clustering (SLIC) and then transforms the features of super pixels using PCA. The transformed features are then used for the final classification (Su, 2019). Herein, PCA was performed on the 10 raw spectral bands for June 2021 using the “Forward PCA Rotation New static and Rotate” tool in ENVI Classic (Table 3). The contribution rate of the first principal component was 98.07%, and the cumulative contribution rate of the first and second principal components was 99.49%, indicating that the first two characteristics accounted for the majority. To ensure computational efficiency, feature extraction with a monthly cumulative contribution rate of 99% was selected, and used the same method to perform PCA on images from July, August, September, and October.

**Table 3:** Principal component analysis results of original bands in each month.

**PC**	**Eigenvalue**	**Percent (%)**
**(a) June**		
1	***********	**98.07**
2	198653.1435	**99.49**
3	50204.4439	99.85
4	7833.7430	99.91
5	5591.6879	99.95
6	3837.2299	99.98
7	1600.1198	99.99
8	823.2481	99.99
9	434.7474	100.00
10	402.0703	100.00
**(b) July**		
1	***********	**92.96**
2	925510.1174	**99.40**
3	54794.9855	99.78
4	10584.4943	99.86
5	7311.4799	99.91
6	6335.4789	99.95
7	3513.3720	99.98
8	1921.6643	99.99
9	945.7515	100.00
10	577.5016	100.00
**(c) August**		
1	***********	**93.61**
2	667177.0382	**99.41**
3	45269.5504	99.78
4	9327.8970	99.86
5	4539.5440	99.91
6	3432.8620	99.95
7	2540.6536	99.98
8	1691.1817	99.99
9	761.6588	100.00
10	352.9345	100.00
**(d) September**		
1	***********	**97.76**
2	178696.0256	**99.35**
3	52125.5981	99.81
4	765.1415	99.88
5	5518.4661	99.93
6	4760.6366	99.97
7	1433.4957	99.98
8	1133.7566	99.99
9	559.6960	100.00
10	487.0376	100.00
**(e) October**		
1	***********	**98.52**
2	102878.8513	**99.36**
3	46063.2139	99.73
4	16295.3402	99.86
5	7186.4223	99.92
6	4443.8691	99.96
7	2203.5974	99.98
8	1118.5134	99.99
9	1064.3939	100.00
10	598.6472	100.00

**** represents Eigenvalue, however, its data digits exceed the range displayed by ENVI, so the software output is ****.

#### Texture Feature Extraction

As far as the texture features of Guyang County are concerned, the most obvious feature was the mountain, of which the coverage was extremely wide, presenting with rough texture features. In addition, the farmland was mainly distributed in relatively flat areas and in a regular state, owing to anthropogenic influences. In contrast, the grassland was distributed in a more scattered pattern and consisted mostly of herbaceous vegetation without a particularly obvious canopy structure. ENVI software was used to extract texture features from the Sentinel-2 imagery of Guyang County, and to calculate the gray level co-occurrence matrix (GLCM), thereby generating a total of eight textures: mean, variance, homogeneity, contrast, dissimilarity, entropy, angular second moment, and correlation (Xu et al., 2016). This allowed for *A. mongholicus* to be better distinguished from the other crops.

#### Classifier Classification

**Figure 6:**
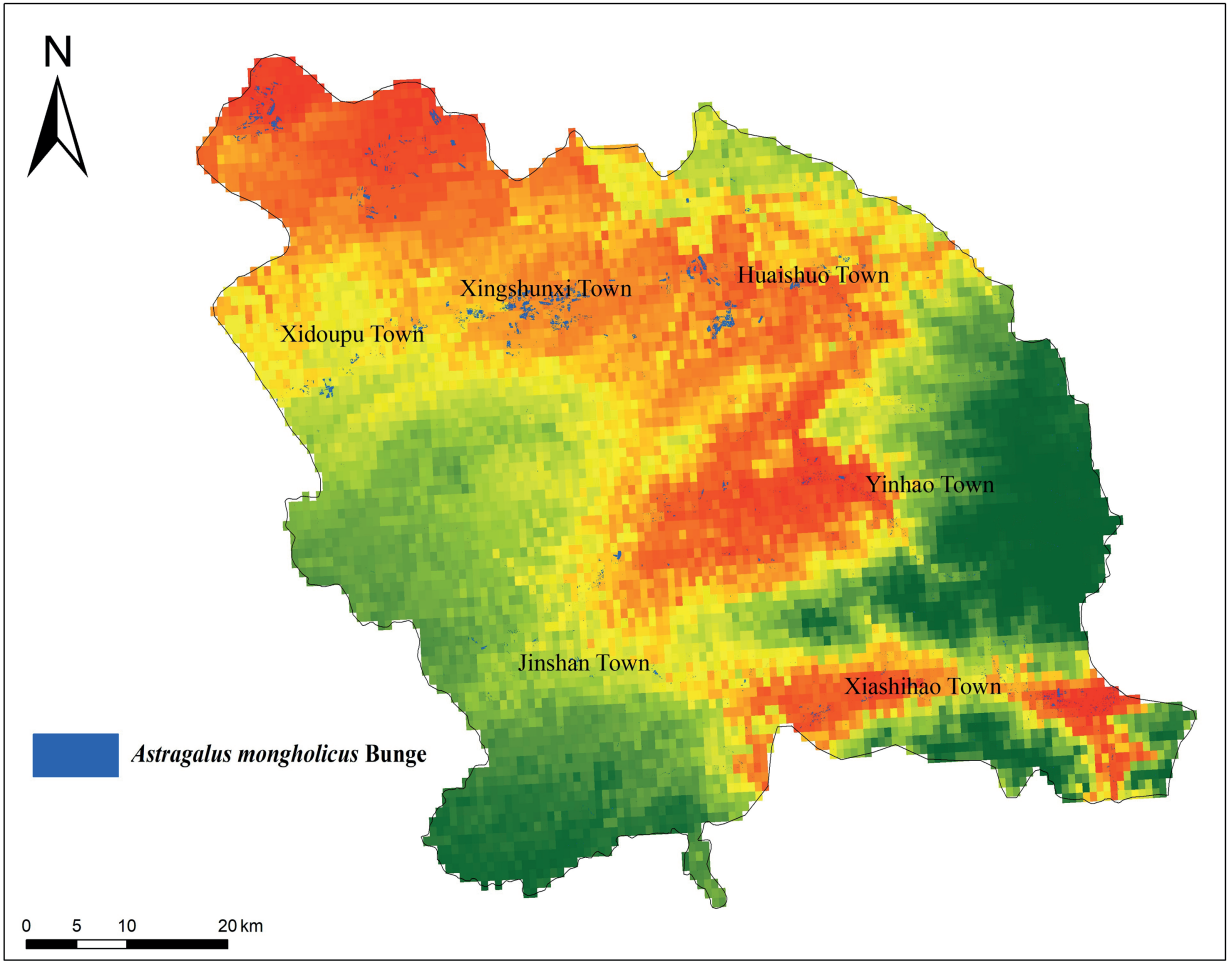
Comparison of random forest prediction results and ecological suitability distribution results.

The classifier was used to extract distribution information of *A. mongholicus*, and the planting area of the evaluated *A. mongholicus* was statistically identified. The sample data were randomly divided into training set (70%) and validation set (30%). The results showed that the actual planting area of Radix Astragali was very consistent with the high suitability growth area predicted by the MaxEnt, and the planting area of *A. mongholicus* in Guyang County was 29.0123 km^2^. Compared with the actual planting area, the accuracy rate was 83.69%, with certain reliability (Figure 6).

#### Accuracy Evaluation

The random forest classification algorithm was used to classify the Sentinel-2 imagery time series including 143 characteristic variables (original spectral feature and vegetation index texture feature), and extracted the distribution information of *A. mongholicus* in the image. The OA of the model was 96.51%, and the Kappa accuracy was 94.96%.

### Industrial Structure and VC

**Figure 7:**
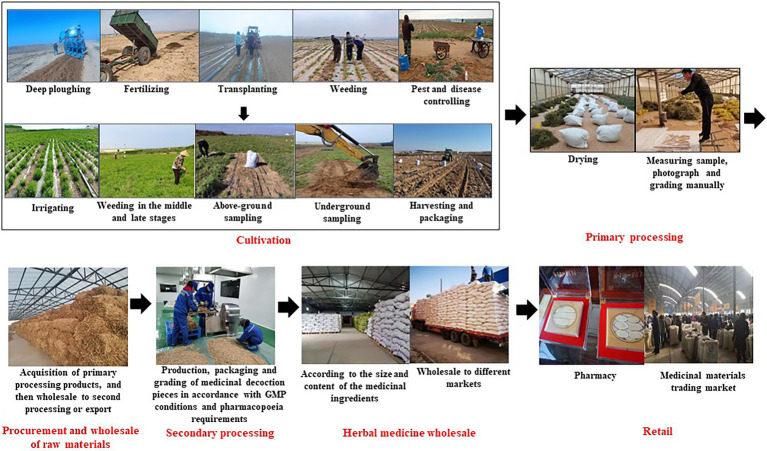
Six stages of *A. mongholicus* before reaching consumers.

As a geographical symbol of agricultural products in China, *A. mongholicus*, a specialty of Guyang County, offers higher productivity and quality. Owing to its long cultivation and supply history, we found that *A. mongholicus* production practices consist of 10 mature VCs, which can be distinguished by their various composite patterns of stakeholders. Typically, *A. mongholicus* undergoes six production stages before it reaches the wholesale and retail herbal markets, as shown in Figure 7.

**Figure 8:**
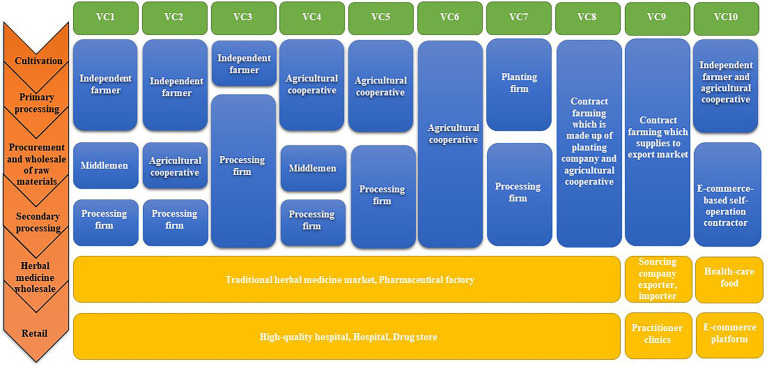
Primary VCs and stakeholders involved in *A. mongholicus* produce.

After conducting the survey, we constructed the VCs, as shown in Figure 8. VCs 1–3 started with independent farmers tending to their own relatively small areas of land to grow *A. mongholicus*. This represents a traditional, small-scale farmer’s economic form, and is an important part of *A. mongholicus* production. In these VCs, farmers usually sell *A. mongholicus* through large suppliers (e.g., middlemen, cooperatives, and processing companies), although the yield of *A. mongholicus* products varies from year to year, which incurs high transaction and switching costs, limiting farmers’ income. However, when farmers are located close to medicinal material markets or processing companies, they can sell their products directly and increase their income, as in VC 3. Most often, middlemen and processing companies finely process and grade the *A. mongholicus* purchased from farmers, subsequently selling it to pharmaceutical factories or Chinese herbal medicine markets to gain higher profits. Finally, the products are retailed to consumers through hospitals, pharmacies, and other avenues. This pattern is most common in Guyang County (Chen et al., 2021).

VCs 4–6 represented agricultural cooperatives with relatively large areas of land, which are usually composed of several farmers who manage large tracts of land through leases or other means, with the capacity to purchase more machinery and equipment than small-scale farmers. These farmers receive more financial, technical, and equipment support to grow *A. mongholicus* individually or cooperatively. Many cooperatives involved in *A. mongholicus* planting tend to sell fresh roots to middlemen or processing companies, thus achieving rapid capital recovery in the second year of planting. In addition, some cooperatives have complete facilities to manage the wholesale, and processing of raw materials. After being subjected to a series of operations, *A. mongholicus* medicinal products in these VCs are obtained and sold directly to the Chinese herbal medicine market and pharmaceutical factories, in the hopes of reducing the consumption cost of middlemen and others, thereby increasing revenue (Jiang et al., 2021).

VC 7 started with a planting company. Planting companies generally cultivate *A. mongholicus* in large plantations using a high degree of mechanization, comprehensive sprinkler irrigation systems, and warehouses storages. After the planting company buys the seeds, it hires farm workers to perform planting, weeding, and pest control. This pattern is more common in the area surrounding Guyang County, where large stretches of land are available for growing *A. mongholicus*.

Cultivation companies in VC 8 were involved in all VC nodes, from production and processing to wholesale. They typically rent the land and hire workers to farm, harvest, pre-process, and then process the *A. mongholicus* in the Companies’ own processing plants. Processed *A. mongholicus* can be sold to hospital pharmacies and private clinics. Because these planting companies simultaneously fulfill the roles of independent farmers, middlemen, and processing entities, VCs involving them are simplified, with reduced costs and improved economic efficiency. Compared with independent farmers and agricultural cooperatives, planting companies have a more standardized planting and processing model. Moreover, all process stages are traceable, including the production chain and planting technology, application of fertilizers and pesticides, and quality inspection. The wholesale and retail sectors can control the quality and supply of *A. mongholicus* products more directly and achieve rational resource allocation and revenue maximization. In addition, most planting companies employ local, experienced farmers for planting and processing, providing job opportunities in the region. These farmers can also find other jobs during the off-season, thereby increasing their income. Planting companies around Guyang County, based on the principles of high-quality standards and timely delivery, sell processed *A. mongholicus* products to wholesale and retail departments, optimizing the supply model of *A. mongholicus* products (Bi et al., 2020).

As an export VC, VC 9 was similar to VC 8 but represented export of *A. mongholicus* products to foreign consumers. In this chain, exported *A. mongholicus* products are subject to stricter quality control measures to improve reliability of the *A. mongholicus* production system and achieve reputation and marketing goals.

VC 10 was an e-commerce-based supply model. With the development of the big data era, the internet-based Chinese medicine trade (e-commerce) is becoming increasingly common, and the future trend entails a shift of the trade center from the market to the place of origin. Contractors (in the form of middlemen or small medical processing plants) are the main players that buy pre-processed *A. mongholicus* directly from independent farmers or agricultural cooperatives, process it into medicinal slices or powder, package it more attractively, and sell it online. In theory, e-commerce should offer higher profits than traditional marketplaces because of significantly lower operating costs; however, the quality control of products sold through online platforms requires significant improvement. Moreover, e-commerce currently represents a relatively new supply model in which sales channels are not yet fully established and sales levels are far lower than those of offline sales. Further in-depth research is required to address these issues.

### Price Volatility and Forecast

In recent years, the price of Chinese herbal medicines has significantly varied at high frequency, resulting in strong uncertainty and intensified market risk. The price data of Mongolian *A. mongholicus* was collected for 5 years and it was evident that the price was not fixed. Price volatility affects all stakeholders in the VCs and has a significant impact on the income for growers. Uncertainty about prices may affect growers’ enthusiasm, resulting in changes in acreage. For wholesalers, price uncertainty can lead to backlogs or reduced revenues. We hope to provide stakeholders with a reference value by predicting future price trends.

**Figure 9:**
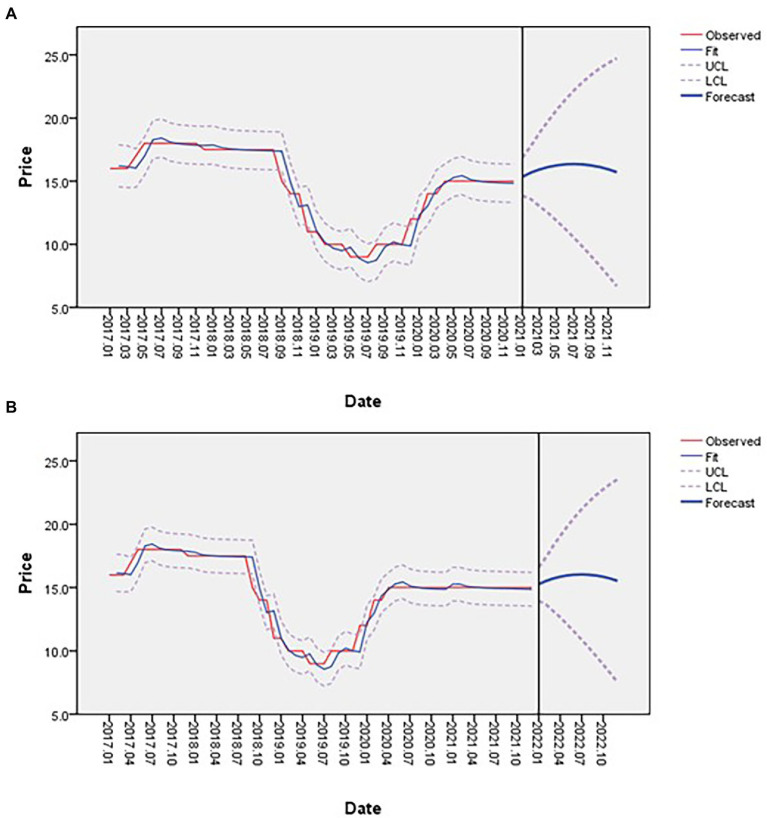
Price forecast for 2021 **(A)**; Price forecast for 2022 **(B)**.

First, we predict the price in 2021 based on the ARIMA model, and get the price of *A. mongholicus* as shown in Figure 9A. The prices of *A. mongholicus* we collected in 2021 are all 15 yuan/kg, and the difference from the predicted value is within the range of 2 yuan/kg, which has a certain accuracy. Meanwhile, the model got a Mean Absolute Percentage Error (MAPE) of 3.369%, and R^2^ was 0.948. Studies have shown that when MAPE is less than 10%, the model fit is better (Nakashima et al., 2021), so the ARIMA price prediction model is feasible.

We predicted the price of *A. mongholicus* in 2022 based on the price from 2017 to 2021, and MAPE was 2.755%, R^2^ was 0.947. Figure 9B shows data collection date in January 2017 (horizontal axis), price of *A. mongholicus* (vertical axis), price forecast trend (blue line), and price forecast confidence interval (dotted line) is depicted. Those prices indicated after January 2022 are forecast prices. During the period from 2017 to September 2018, the price of *A. mongholicus* was relatively stable. However, during September 2018–May 2020, the price of *A. mongholicus* experienced a rapid decline and recovery. After May 2020, the price has remained stable at 15 yuan/kg. In 2022, the price of *A. mongholicus* has a gentle rise and gradually stabilized.

According to the ARIMA price forecast model, the market price of *A. mongholicus* in Mongolia may be expected to show a steady future trend, although the overall impact may not be significant. This can be gradually improved by adjusting the supply relationship related to *A. mongholicus* production, processing, and sales, thereby alleviating drastic price fluctuations.

## Discussion

### Practical Application of SDM Prediction

The quality of medicinal plant products can be more clearly identified in different markets by understanding the internal and external linkages between production, processing, and trade networks. The added value offered by medicinal plants can be introduced at various stages of plant production. As the first step in its VC, *A. mongholicus* cultivation techniques are essential to increasing its value. In our study, the ecological and quality suitability of *A. mongholicus* was partitioned, and the quality of this medicinal plant was closely related to the choice of cultivation site. Therefore, in the process of converting medicinal plant raw materials into high-value products, the realization of value depends not only on actual production, but also on scientific guidance, which is an indispensable step (Yang et al., 2020a).

A high overlap was found between the suitable distribution area of *A. mongholicus* and the area with the highest active components. However, the environmental data that has the greatest impact on the suitable growth of *A. mongholicus* was the precipitation in the warmest quarter, although it was not the most important environmental data to promote the accumulation of its effective components. In short, although there were many similarities between the suitable distribution area and the high-quality distribution area of *A. mongholicus*, the ecological and environmental factors affecting the two were different. Studies have shown that the promotion of secondary metabolite accumulation in medicinal plant tissues is related to the interactions of multiple environmental factors (Jiang et al., 2020). The quality of many authentic medicinal plants is related to certain environmental stressors, and it is proposed that the formation of high-quality medicinal materials may need to experience unfavorable environmental conditions (Huang and Guo, 2007) Therefore, further research is needed to explore the influence of the environment on the distribution of high-quality medicinal plants. However, the contribution of altitude to the study was not significant. It may be that most of *A. mongholicus* in Guyang County was planted in flat and broad agricultural land. The altitude range of *A. mongholicus* planting in the whole county was similar, thus the altitude had little effect. Whether altitude has an impact on the growth of *A. mongholicus* needs further study in an area with large altitude differences.

In this study, the predicted distribution of SDMs was similar to the distribution extracted from remote sensing, reflecting the major potential of using geographic information to predict the distribution of *A. mongholicus*. Furthermore, the feasibility of employing SDMs to guide the introduction and cultivation of medicinal plants was demonstrated. In a previous study, Wang et al. (2021b) used MaxEnt to estimate the impact of climate change on the distribution of potatoes in China from 1961 to 2017, predicting its suitable planting areas and guiding its planting distribution (Wang et al., 2021b).

### Practical Application of Remote Sensing Prediction

Based on the Sentinel-2 imagery, the planting distribution information of *A. mongholicus* in Guyang County was extracted and the distribution and planting area of *A. mongholicus* was obtained. This method had high feasibility and can aid government departments and farmers to make informed production decisions. In particular, government departments can identify discrepancies by comparing remotely sensed planting data to those reported by farmers or businesses. In a previous study, we used ZY-3 satellite imagery as a remote sensing data source to interpret the Saposhnikovia planting situation in Naiman Banner. Thereafter, 20 sample plots were randomly selected for field measurement, and the accuracy was evaluated by comparisons with the interpretation results. Subsequent calculations revealed the accuracy of extracting the Saposhnikovia planting area at 93.90%, which meets the requirements of remote sensing monitoring of agricultural conditions (Jia et al., 2019). Remote sensing technology is widely used in agriculture; however, in the investigation of medicinal plant resources, cultivated medicinal plants are mainly monitored, whereas wild medicinal plants are rarely monitored, and a lot of experimental verifications thus remain necessary. Subsequent studies should further monitor the yield, diseases, and insect pests of medicinal plants, so as to provide more statistical data for relevant management departments and promote the formulation of relevant policies and stable economic development.

### The Relationship Between Behavior, Income, and Quality

In traditional markets, VC stakeholders are inclined to take steps toward cost reduction and profit growth, however, this can lead to reduced product quality. VC 1 and 2, constructed herein, were typical examples of traditional markets. To achieve high yield in such VCs, excessive chemical drugs may have been used without professional guidance, and unreasonable planting measures may have been implemented, resulting in reduced quality and safety of *A. mongholicus*. Therefore, quality issues were common in these ventures, although the income of farmers was relatively low.

VCs 3–6 reflected relatively standardized market models involving larger acreages and more standardized planting patterns than that of independent farmers. However, they were highly variable and determined the quality of a medicinal materials based on the market environment.

In high-quality markets, companies were more comprehensively involved with some form of self-regulation. Consequently, their brand reputations were improved, thereby increasing their overall value. The production in VCs 7 and 8 reflected a high-quality product that would enter a high-quality market. Quality products are key to the development of a factory, and the reliability and traceability of its products allows it to build a good reputation.

In the export market, relevant companies strictly controlled every step of the production process and extensive quality tracking of *A. mongholicus* was performed by conducting germplasm selection and soil testing in the planting area. As such, it was ensured that all aspects (from planting to product packaging), met the inspection standards required by foreign markets. VC 9 implemented effective quality control measures during cultivation and production, resulting in the production of high-quality *A. mongholicus*. This would result in highly lucrative returns for the stakeholders. Selling medicinal materials through e-commerce platforms, as in VC 10, could greatly reduce store rental costs. However, several gaps remained in the supervision process; high-quality medicinal materials from the same source could be classified into different grades and enter the market through different channels. Therefore, the behavior and interests of stakeholders, as well as the quality and target market of the product, were closely related.

The Chinese herbal medicine industry has a long chain with a wide range and many limitations. It is necessary to consider the information associated with its cultivation, processing, acquisition, storage, transportation, and sales in addition to paying more attention to the drug properties of Chinese herbal medicines. Moreover, guidance practices on the development of the Chinese herbal medicine industry should be strengthened, the large fluctuation of total output and prices caused by production dispersion should be alleviated, and the establishment of price formation mechanisms oriented by high quality and high prices should be promoted. The Chinese herbal medicine industry is highly focused on authenticity. Unlike most crops, which focus on production yield, Chinese herbal medicines are more oriented toward quality assurance. Therefore, the production of Chinese herbal medicines required unique developmental directions and ideas.

### Price Fluctuation Factors

Along with improvement on the economic level, people pay increasingly more attention to their own health care and disease prevention. Market price fluctuations of traditional Chinese medicines are not only closely related to its production costs, but are also affected by factors such as policies, climate, and epidemics.

Traditional Chinese medicine plays an active role in the early interventional treatment of diseases. Moreover, according to existing literature and clinical experience, Chinese herbal medicine has a therapeutic effect on COVID-19 (Leung et al., 2020). These products have become an important part of China’s fight against the pandemic, resulting in an increased demand for Chinese herbal medicine. Under the current state of prevention and control of viral dissemination, it may be difficult to harvest Chinese herbal medicines and its transportation may be inconvenienced in some areas, resulting in price fluctuations. In addition, due to the impact of bad weather patterns, drought and flood disasters have reduced production in some areas, and the worldwide spread of the epidemic has also led to insufficient supply of imports and exports (Wu and He, 2021). Overall, the demand for Chinese herbal medicines has increased; however, supply has decreased, which inevitably leads to price fluctuations. Nevertheless, it is worth noting that the planting area of Chinese herbal medicines has annually increased in recent years, which would likely improve the supply and demand problem to a certain extent and alleviate the sharp price fluctuations.

### Limitations of This Study

In this study, we selected Guyang County as the study area, considering that the *A. mongholicus* grown in this area has been recognized as an agricultural geographical indication product by the Ministry of Agriculture and Rural Affairs of the People’s Republic of China, and the high quality of *A. mongholicus* grown in this area has been recognized by the state and society. However, Guyang County is small, the *A. mongholicus* cultivation area is limited, and the relationship between supply and demand cannot be improved to a large extent. In order to effectively explore the suitable planting areas of *A. mongholicus* in the country, we executed a detailed investigation of various data. According to the results of the fourth census of traditional Chinese medicine resources, there exists a large number of *A. mongholicus* in Northeast, North, and Northwest China, especially in Shanxi and Gansu. Nevertheless, this study provides a reference for future research.

## Conclusion

With increasing demand for traditional Chinese medicine in China, the sustainable development of the traditional Chinese medicine industry has received more attention. In this study, we combined remote sensing technology with SDM to predict the suitable growth area of *A. mongholicus* in Guyang County, aiming to provide theoretical guidance for the selection of *A. mongholicus* planting areas. The relationship model between the active components and ecological factors of *A. mongholicus* in Guyang County was established to evaluate the main factors affecting the accumulation of active components in *A. mongholicus*. Among these ecological factors, mean temperature of the coldest quarter (BIO11) played an important role in the accumulation of astragaloside IV. Moreover, mean diurnal range [mean of monthly (max temp - min temp)] (BIO2) and precipitation of the coldest quarter (BIO19) influenced the accumulation of calycaryin-7-glucoside. The northern and central regions of Guyang County were predicted to be suitable planting areas for high-quality *A. mongholicus*. Sentinel-2 imagery were used to monitor the growth area of *A. mongholicus*, which resembled the actual planting situation and provided data references which may be used by the relevant management departments to formulate the required policies and plans for conducting economic management. Moreover, the behavior of stakeholders, suitability of geographic planting areas, and target markets had significant influences on the quality of *A. mongholicus*. Stakeholders in each VC played different roles in the cultivation, processing, and sales of *A. mongholicus*. However, in addition to the selected planting area and associated quality, yield, pests, and diseases also affected the supply of *A. mongholicus*. In future research, remote sensing technology should be used to monitor the yield, diseases, and insect pests of *A. mongholicus*, balance supply and demand, and aid in the development of the *A. mongholicus* industry. Our research proved that it is necessary to conduct a larger-scale and more comprehensive study on the suitable cultivation areas of *A. mongholicus* by means of remote sensing and SDM, to lay a foundation for deepening its role in the environment and to allow for its sustainable utilization.

With the rise of the trend of advocating traditional Chinese medicine and natural medicine in the world, the international recognition of medicines and health food produced from natural Chinese herbal medicine resources has been increasing, and the development and utilization of Chinese herbal medicine resources has become an important trend in the development of medicine in the world. As a health care product, *A. mongholicus* can be used both as medicine and as food, and has a wide range of uses. In order to give full play to the role of *A. mongholicus*, in addition to continuing to strengthen planting techniques, we should also develop *A. mongholicus* products that meet different needs according to the uniqueness of *A. mongholicus*. Exploiting its market potential is of great significance to promoting the development of *A. mongholicus* industry, promoting regional development, and building a green *A. mongholicus* production base.

## Data Availability Statement

The datasets presented in this study can be found in online repositories. The names of the repository/repositories and accession number(s) can be found in the article/Supplementary Material.

## Author Contributions

RZ conducted the experiment and analyzed the data. MZ, YC, YY, LJ, and XW conducted the experiment. XZ, HL, and ML designed the experiment. All authors have reviewed the final version and approved it for publication.

## Funding

This work was supported by Ministry of Agriculture and Rural Affairs of the People’s Republic of China (Grant number: CARS-21); National Administration of Traditional Chinese medicine [Grant number: Finance Society (2019), 39]; National Natural Science Foundation of China (Grant number: M1942003); Inner Mongolia Mongolian Medicine Standardization Project [2020-(MB015)]; and Science and technology program in Inner Mongolia (Grant number: 2020GG0144).

## Conflict of Interest

The authors declare that the research was conducted in the absence of any commercial or financial relationships that could be construed as a potential conflict of interest.

## Publisher’s Note

All claims expressed in this article are solely those of the authors and do not necessarily represent those of their affiliated organizations, or those of the publisher, the editors and the reviewers. Any product that may be evaluated in this article, or claim that may be made by its manufacturer, is not guaranteed or endorsed by the publisher.

## Footnotes

^1^ http://www.moa.gov.cn/nybgb/2017/dsq/201802/t20180201_6136210.htm

^2^ http://www.aqsc.agri.cn/tzgg/202009/t20200918_361324.htm

^3^ http://www.worldclim.org/

^4^ https://scihub.copernicus.eu/

## References

[ref1] Alabdulrazzaq H. Alenezi M. N. Rawajfih Y. Alghannam B. A. Al-Hassan A. A. Al-Anzi F. S. (2021). On the accuracy of ARIMA based prediction of COVID-19 spread. Results Phys. 27:104509. doi: 10.1016/j.rinp.2021.104509, PMID: 34307005 PMC8279942

[ref2] Bi Y. Q. Bao H. Y. Zhang C. H. Yao R. Y. Li M. H. (2020). Quality control of Radix Astragali (The root of Astragalus membranaceus var. mongholicus) Along its value chains. Front. Pharmacol. 11:562376. doi: 10.3389/FPHAR.2020.562376, PMID: 33343346 PMC7746871

[ref3] Booker A. Johnston D. Heinrich M. (2012). Value chains of herbal medicines Rresearch needs and key challenges in the context of Ethnopharmacology. J. Ethnopharmacol. 140, 624–633. doi: 10.1016/j.jep.2012.01.039, PMID: 22326378

[ref4] Booker A. Johnston D. Heinrich M. (2015). Value chains of herbal medicines Ethnopharmacological and analytical challenges in a globalizing world. Evi. Validation Herb. Med. 29–44. doi: 10.1016/B978-0-12-800874-4.00002-7

[ref5] Breiman L. (2001). Random forests. Mach. Learn. 45, 5–32. doi: 10.1023/A:1010933404324

[ref6] British Pharmacopoeia Commission (2019). British Pharmacopoeia (Herbal Drug Preparations and Herbal Medicinal Products). England: The Stationery Office.

[ref7] Chen X. (2021). Application of satellite remote sensing Technology in Forestry Investigation and Planning. Modern Agri. Res. 27, 79–80. doi: 10.19704/j.cnki.xdnyyj.2021.08.037

[ref8] Chen Y. Lei L. J. Bi Y. Q. Jiang L. L. Guo W. F. Wang J. H.. (2021). Quality control of Glehniae Radix, the root of *Glehnia Littoralis* Fr. Schmidt ex Miq., Along its Value Chains. Front. Pharmacol. 12:729554. doi: 10.3389/FPHAR.2021.729554, PMID: 34671256 PMC8521048

[ref10] Cui X. S. Ding Y. H. Gao X. Q. Jin P. B. Guo Y. H. Dong X. H. (2020). Empirical study on market Price classification and prediction of traditional Chinese medicine based on multiple linear regression model. Northern Hortic. 14, 157–161. doi: 10.11937/bfyy.20193701

[ref11] Durazzo A. Nazhand A. Lucarini M. Silva A. M. Souto S. B. Guerra F.. (2021). Astragalus (astragalus membranaceus bunge): botanical, geographical, and historical aspects to pharmaceutical components and beneficial role. Rendiconti Lincei Sci. Fisiche Nat. 32, 625–642. doi: 10.1007/s12210-021-01003-2

[ref12] European Pharmacopoeia Commission (2017). European Pharmacopoeia. Strasbourg: European Directorate for the Quality of Medicines and Health Care.

[ref13] George F. J. Fred C. C. (1971). Error on Choroplethic maps: definition, measurement, reduction. Ann. Assoc. Am. Geogr. 61, 217–244. doi: 10.1111/j.1467-8306.1971.tb00779.x

[ref14] Hess A. S. Hess J. R. (2018). Principal component analysis. Transfusion 58, 1580–1582. doi: 10.1111/trf.14639 29732564

[ref15] Huang L. Q. Guo L. P. (2007). Formation of medicinal herbs. J. Chinese Mater. Med. 32, 277–280. doi: 10.3321/j.issn:1001-5302.2007.04.001

[ref16] Inoue Y. Olioso A. (2006). Estimating the dynamics of ecosystem co2flux and biomass production in agricultural fields on the basis of synergy between process models and remotely sensed signatures. J. Geophys. Res. 111:D24. doi: 10.1029/2006jd007469

[ref17] Jaynes E. T. (1957). Information theory and statistical mechanics. Phys. Rev. 106, 620–630. doi: 10.1103/PhysRev.106.620

[ref18] Jia J. Y. Cao R. Zhang X. B. Shi T. T. Yang M. Li M. H. (2019). Monitoring of Saposhnikovia divaricate planting area based on texture and pop information in Naiman banner. China J. Chinese Mater. 44, 4111–4115. doi: 10.19540/j.cnki.cjcmm.20190731.111, PMID: 31872685

[ref19] Jiang D. Q. Wang H. Y. Kang C. Z. Jiang J. Y. Du Y. X. Zhang Y.. (2020). Influence and mechanism of stress combination on medicinal plants secondary metabolism. China J. Chinese Mater. Med. 45, 2009–2016. doi: 10.19540/j.cnki.cjcmm.20200302.106, PMID: 32495546

[ref20] Jiang L. L. Zhou B. C. Wang X. Q. Bi Y. Q. Guo W. F. Wang J. H.. (2021). The quality monitoring of Cistanches Herba (*Cistanche deserticola* Ma): A value chain perspective. Front. Pharmacol. 12:782962. doi: 10.3389/FPHAR.2021.782962, PMID: 34803722 PMC8602053

[ref21] Lan J. X. Zhang F. Lian C. L. Li C. (2021). Application of remote sensing Technology in Medicinal Plant Resources. Technol. Innov. App. 11, 167–170. 10.19540/j.cnki.cjcmm.20210620.101 34581077

[ref22] Leung E. L. Pan H. D. Huang Y. F. Fan X. X. Wang W. Y. He F.. (2020). The scientific foundation of chinese herbal medicine against COVID-19. Engineering 6, 1099–1107. doi: 10.1016/j.eng.2020.08.009, PMID: 33520331 PMC7833648

[ref23] Liu Y. T. Li Z. Y. Li H. K. Wang X. L. (2021). A southern citrus woodland extraction method combing phenological and texture features. Sci. Surveying Mapping 46, 83–93. doi: 10.16251/j.cnki.1009-2307.2021.09.011

[ref24] Na R. H. Zheng J. H. Guo B. L. Sen B. T. Shi M. H. Sun Z. Q.. (2013). Remote sensing estimation of safflower planting area based on PCA and texture features. J. Chinese Mater. 38, 3681–3686. doi: 10.4268/cjcmm20132116 24494554

[ref25] Nakashima T. Ogata S. Noguchi T. Tahara Y. Onozuka D. Kato S.. (2021). Machine learning model for predicting out-of-hospital cardiac arrests using meteorological and chronological data. Heart 107, 1084–1091. doi: 10.1136/heartjnl-2020-318726, PMID: 34001636 PMC8223656

[ref26] National Pharmacopoeia Committee (2020). Committee for the Pharmacopoeia of PR China. Pharmacopoeia of PR China (Part I). Beijing: People’s Health Publishing.

[ref27] Ngarega B. K. Masocha V. F. Harald S. (2021). Forecasting the effects of bioclimatic characteristics and climate change on the potential distribution of *Colophospermum mopane* in southern Africa using maximum entropy (MaxEnt). Eco. Inform. 65:101419. doi: 10.1016/j.ecoinf.2021.101419

[ref28] Phillips S. J. Anderson R. P. Schapire R. E. (2006). Maximum entropy modeling of species geographic distributions. Ecol. Model. 190, 231–259. doi: 10.1016/j.ecolmodel.2005.03.026

[ref29] Qi X. M. (2020). Analysis of common problems and countermeasures in planting of *Astragalus mongholicus* Bunge. Agri. Dev. Equip. 161–162. doi: 10.3969/j.issn.1673-9205.2020.11.077

[ref30] Qin X. M. Li Z. Y. Sun H. F. Zhang L. Z. Zhou R. Feng Q. J.. (2013). Current situation and analysis of Astragalus medicinal materials resources in my country. J. Chin. Mater. 38, 3234–3238.

[ref31] Rao J. N. K. Box G. E. P. Jenkins G. M. (1972). Time Series Analysis Forecasting and Control. Econometrica 40, 970–971. doi: 10.2307/1912100

[ref32] Rural Social and Economic Investigation Department of National Bureau of Statistics (2021). China Statistical Yearbook (County-level). Beijing: China Statistics Press.

[ref33] Shi T. T. Zhang X. B. Guo L. P. Huang L. Q. (2017). Study of extracting Peucedanum praeruptorum planted area in Ningguo of Anhui province based on multi-source and multi-phase image. J. Chin. Mater. 42, 4362–4367. doi: 10.19540/j.cnki.cjcmm.2017.0185, PMID: 29318836

[ref34] Su T. F. (2019). Superpixel-based principal component analysis for high resolution remote sensing image classification. Multimedia tools and applications. Multimed. Tools Appl. 78, 34173–34191. doi: 10.1007/s11042-019-08224-6

[ref35] Sun S. Y. Chen G. L. (2018). Current situation, problems and countermeasures of Radix astragali industrialization in Inner Mongolia. Mol. Plant Breeding. 16, 5126–5133. doi: 10.13271/j.mpb.016.005126

[ref36] Tan S. X. Johnson S. Gu Z. (2018). Laser depolarization ratio measurement of corn leaves from the biochar and non-biochar applied plots. Opt. Express 26:14295. doi: 10.1364/oe.26.014295, PMID: 29877470

[ref37] Vasileios S. Ioannis P. Charalampos K. Alberto A. Andrés A. P. Zurbano J. A. (2018). Scalable parcel-based crop identification scheme using Sentinel-2 data time-series for the monitoring of the common agricultural policy. Remote Sens. 10:911. doi: 10.3390/rs10060911

[ref38] Wang C. Shi X. Y. Liu J. G. Zhao J. C. Bo X. Z. Chen F.. (2021a). Interdecadal variation of potato climate suitability in China. Agric. Ecosyst. Environ. 310:107293. doi: 10.1016/j.agee.2020.107293

[ref39] Wang Z. J. Wei M. S. Guo L. F. Wang X. Q. Gao L. (2021b). Extraction of crop spatial distribution information based on principal component analysis. Geomatics & Spatial Information. Technology 44, 114–119. doi: 10.3969/j.issn.1672-5867.2021.06.031

[ref40] Wu W. X. He L. Y. (2021). The general increase in the price of Chinese medicinal materials continues, and the northern flood has become a major incentive. 21st Century Bus. Herald. 6, 1–2. doi: 10.28723/n.cnki.nsjbd.2021.004624

[ref41] Xu X. Li J. Huang X. Mura M. D. Plaza A. (2016). Multiple morphological component analysis based decomposition for remote sensing image classification. IEEE Trans. Geosci. Electron. 54, 3083–3102. doi: 10.1109/TGRS.2015.2511197

[ref42] Xu Z. L. Peng H. H. Peng S. Z. (2015). The development and evaluation of species distribution models. Acta Ecol. Sin. 35, 557–567. doi: 10.5846/stxb201304030600

[ref43] Yang M. Li Z. Y. Liu L. B. Bo A. Zhang C. H. Li M. H. (2020a). Ecological niche modeling of *Astragalus membranaceus* var. mongholicus medicinal plants in Inner Mongolia. China. Sci. Rep. 10:12482. doi: 10.1038/s41598-020-69391-3, PMID: 32719330 PMC7385632

[ref44] Yang B. Zhang Q. J. Wang B. Gong X. Zhang Y. B. (2020b). The habitat suitability evaluation of *Salweenia bouffordiana* based on MaxEnt model. Acta Ecol. Sin. 40, 6077–6085. doi: 10.5846/stxb201906031167

[ref45] Yao R. Y. Heinrich M. Wang Z. G. Weckerle C. (2018). Quality control of goji (fruits of *Lycium barbarum* L. and *L. chinense* mill.): A value chain analysis perspective. J. Ethnopharmacol. 224, 349–358. doi: 10.1016/j.jep.2018.06.010, PMID: 29908314

[ref46] Zhang M. X. Jiang D. Yang M. Ma T. Ding F. Y. Hao M. M.. (2021). Influence of the environment on the distribution and quality of Gentiana dahurica Fisch. Front. Plant Sci. 12:706822. doi: 10.3389/fpls.2021.706822, PMID: 34646283 PMC8503573

[ref47] Zhao Y. S. Li Z. Y. Na M. H. Han Q. H. Ren K. Zhang C. H.. (2017). Analysis and suggestion of ecological planting status about Chinese-Mongolian traditional medicine in Inner Mongolia. Modern Chin. Med. 19, 901–906. doi: 10.13313/j.issn.1673-4890.2017.7.002

[ref48] Zhu G. P. Liu G. Q. Bu W. J. Gao Y. B. (2013). Ecological niche modeling and its applications in biodiversity conservation. Biodivers. Sci. 21, 90–98. doi: 10.3724/SP.J.1003.2013.09106

[ref49] Zhu S. D. Peng H. S. Guo L. P. Xu T. R. Zhang Y. Chen M. L.. (2017). Regionalization of Chinese material medical quality based on maximum entropy model: A case study of *Atractylodes lancea*. Sci. Rep. 7:42417. doi: 10.1038/srep42417, PMID: 28205539 PMC5311955

