# Supplementary material for: Promoting the Development of Astragalus mongholicus Bunge Industry in Guyang County (China) Based on MaxEnt and Remote Sensing
Source: Front Plant Sci. 2022 Jul 7;13:908114. doi: 10.3389/fpls.2022.908114 (PMC9301113; doi:10.3389/fpls.2022.908114)
Supplement: Supplementary file 1 [file Table_1.DOCX]

Supplementary Material

Promoting the Development of *Astragalus mongholicus* Bunge Industry in Guyang County (China) Based on Maxent and Remote Sensing

Ru Zhang^1^, Mingxu Zhang^1^, Yumei Yan^1^, Yuan Chen^4^, Linlin Jiang^4^, Xinxin Wei^5^, Xiaobo Zhang^6^, Huanting Li^1*^ and Minhui Li^1,2,4,5*^

^1^Baotou Medical College, Baotou, Inner Mongolia, China

^2^Inner Mongolia Autonomous Region Hospital of Traditional Chinese medicine, Hohhot, Inner Mongolia, China

^3^Inner Mongolia Key Laboratory of Characteristic Geoherbs Resources Protection and Utilization, Baotou, Inner Mongolia, China

^4^Inner Mongolia Medical University, Hohhot, Inner Mongolia, China

^5^Inner Mongolia University, Hohhot, China

^6^State Key Laboratory Breeding Base of Dao-di Herbs, National Resource Center for Chinese Materia Medica, China Academy of Chinese Medical Sciences, Beijing, China

*** Correspondence:**Minhui Li
[prof_liminhui@yeah.net](mailto:prof_liminhui@yeah.net)

Huanting Li
[lihuanting2021@163.com](mailto:lihuanting2021@163.com)

# Supplementary Figure 1 Geographic information of *Astragalus mongholicus* Bunge samples and determination results of the active components of 37 samples

| NO. | longitude | latitude | calycosin-7-glucoside（%） | astragaloside-IV（%） |
| --- | --- | --- | --- | --- |
| S1 | 109.818859 | 41.400692 | 0.0725 | 0.08635 |
| S2 | 109.675775 | 41.443248 | 0.017 | 0.1647 |
| S3 | 109.659498 | 41.451427 | 0.024274205 | 0.1426 |
| S4 | 109.668523 | 41.468353 | 0.0503 | 0.1402 |
| S5 | 109.789915 | 41.358611 | 0.0515 | 0.07065 |
| S6 | 109.833228 | 41.418719 | 0.0241 | 0.08595 |
| S7 | 109.790758 | 41.36391 | 0.0347 | 0.1109 |
| S8 | 109.819156 | 41.356172 | 0.0235 | 0.105 |
| S9 | 110.323412 | 41.291123 | 0.027 | 0.1227 |
| S10 | 110.310267 | 41.307732 | 0.0396 | 0.12525 |
| S11 | 110.324001 | 41.289899 | 0.0557 | 0.1793 |
| S12 | 110.311095 | 41.103461 | 0.0254 | 0.193 |
| S13 | 110.320048 | 41.112242 | 0.0596 | 0.0878 |
| S14 | 110.383381 | 41.085599 | 0.0499 | 0.14055 |
| S15 | 110.583923 | 40.902454 | 0.031 | 0.1327 |
| S16 | 110.581366 | 40.903753 | 0.0764 | 0.1131 |
| S17 | 110.583761 | 40.903155 | 0.0423 | 0.17825 |
| S18 | 110.64472 | 40.906232 | 0.0768 | 0.10605 |
| S19 | 109.973317 | 41.245539 | 0.049 | 0.1587 |
| S20 | 109.975665 | 41.238929 | 0.0145 | 0.1496 |
| S21 | 109.976405 | 41.235256 | 0.0365 | 0.17425 |
| S22 | 110.223227 | 40.942485 | 0.0422 | 0.1183 |
| S23 | 110.5595 | 41.030546 | 0.0152 | 0.0946 |
| S24 | 110.558498 | 41.030315 | 0.0184 | 0.08185 |
| S25 | 110.559356 | 41.031469 | 0.0479 | 0.10095 |
| S26 | 110.350112 | 41.30152 | 0.0174 | 0.32075 |
| S27 | 110.521694 | 40.902542 | 0.0537 | 0.084 |
| S28 | 110.050192 | 41.00845 | 0.0527 | 0.084 |
| S29 | 110.549964 | 40.907797 | 0.106 | 0.0981 |
| S30 | 109.893119 | 41.274792 | 0.0428 | 0.04454 |
| S31 | 110.5976 | 40.911525 | 0.0663 | 0.115 |
| S32 | 110.337203 | 41.196561 | 0.0258 | 0.1065 |
| S33 | 110.325775 | 41.111833 | 0.0812 | 0.0538 |
| S34 | 110.308322 | 41.2901 | 0.0239 | 0.0342 |
| S35 | 110.594167 | 40.891111 | 0.082 | 0.0121 |
| S36 | 110.595649 | 40.889009 | 0.0189 | 0.0698 |
| S37 | 110.601948 | 40.89702 | 0.0449 | 0.046 |
| S38 | 110.3815583 | 40.91660833 |  |  |
| S39 | 110.3815278 | 40.91665556 |  |  |
| S40 | 110.3811944 | 40.91686944 |  |  |
| S41 | 110.3805778 | 40.90971389 |  |  |
| S42 | 110.3802056 | 40.90988333 |  |  |
| S43 | 110.3804972 | 40.91003611 |  |  |
| S44 | 110.3804167 | 40.91465278 |  |  |
| S45 | 110.4060861 | 40.92261111 |  |  |
| S46 | 110.4076361 | 40.922825 |  |  |
| S47 | 110.4118528 | 40.923275 |  |  |
| S48 | 110.4162444 | 40.917125 |  |  |
| S49 | 110.5071167 | 40.91534722 |  |  |
| S50 | 110.6513056 | 40.89958611 |  |  |
| S51 | 110.6511 | 40.89950278 |  |  |
| S52 | 110.6485806 | 40.89368333 |  |  |
| S53 | 110.6485639 | 40.89369167 |  |  |
| S54 | 110.6473917 | 40.89296944 |  |  |
| S55 | 110.6406528 | 40.88796389 |  |  |
| S56 | 110.6410028 | 40.889275 |  |  |
| S57 | 110.6464333 | 40.88838056 |  |  |
| S58 | 110.6411306 | 40.88899167 |  |  |
| S59 | 110.6416194 | 40.88917778 |  |  |
| S60 | 110.6440972 | 40.89116667 |  |  |
| S61 | 110.6442333 | 40.891075 |  |  |
| S62 | 110.6471194 | 40.89222222 |  |  |
| S63 | 110.6512583 | 40.897225 |  |  |
| S64 | 110.6446278 | 40.90584722 |  |  |
| S65 | 110.6443222 | 40.90615833 |  |  |
| S66 | 110.6321861 | 40.89677222 |  |  |
| S67 | 110.5931639 | 40.89983611 |  |  |
| S68 | 110.5891611 | 40.90001389 |  |  |
| S69 | 110.5888056 | 40.89888889 |  |  |
| S70 | 110.5671083 | 40.904875 |  |  |
| S71 | 110.5640889 | 40.90441389 |  |  |
| S72 | 110.5562694 | 40.904775 |  |  |
| S73 | 110.5493278 | 40.90219444 |  |  |
| S74 | 110.5508139 | 40.90161667 |  |  |
| S75 | 110.5507083 | 40.89995 |  |  |
| S76 | 110.5657667 | 40.89466667 |  |  |
| S77 | 110.5658833 | 40.89481667 |  |  |
| S78 | 110.5676361 | 40.89519444 |  |  |
| S79 | 110.5367361 | 40.90861389 |  |  |
| S80 | 110.5365889 | 40.90871389 |  |  |
| S81 | 110.5295444 | 40.91046667 |  |  |
| S82 | 110.5217417 | 40.91426389 |  |  |
| S83 | 110.6342306 | 40.89628333 |  |  |
| S84 | 110.6338861 | 40.89585 |  |  |
| S85 | 110.6334806 | 40.89528889 |  |  |
| S86 | 110.6376833 | 40.89803333 |  |  |
| S87 | 110.6243278 | 40.90043333 |  |  |
| S88 | 110.607575 | 40.89920556 |  |  |
| S89 | 110.5963972 | 40.8939 |  |  |
| S90 | 110.5964917 | 40.89317778 |  |  |
| S91 | 110.5909361 | 40.89605833 |  |  |
| S92 | 110.5903917 | 40.89979722 |  |  |
| S93 | 110.5908028 | 40.89996944 |  |  |
| S94 | 110.5877667 | 40.90016111 |  |  |
| S95 | 110.5816 | 40.90521944 |  |  |
| S96 | 110.5646611 | 40.90417778 |  |  |
| S97 | 110.5554083 | 40.90482222 |  |  |
| S98 | 110.5506167 | 40.902925 |  |  |
| S99 | 110.5517972 | 40.90240833 |  |  |
| S100 | 110.5505222 | 40.90071667 |  |  |
| S101 | 110.5656944 | 40.89497778 |  |  |
| S102 | 110.567925 | 40.89553889 |  |  |
| S103 | 110.5796694 | 40.89999444 |  |  |
| S104 | 110.5294611 | 40.91027778 |  |  |
| S105 | 110.5299556 | 40.91239444 |  |  |
| S106 | 110.52125 | 40.914 |  |  |
| S107 | 109.8783417 | 41.42558056 |  |  |
| S108 | 109.8856889 | 41.42844167 |  |  |
| S109 | 109.886025 | 41.42809167 |  |  |
| S110 | 109.8893833 | 41.42941389 |  |  |
| S111 | 109.9059611 | 41.42824444 |  |  |
| S112 | 109.9061333 | 41.42830833 |  |  |
| S113 | 109.9059833 | 41.42768056 |  |  |
| S114 | 109.8936972 | 41.43425 |  |  |
| S115 | 109.8934611 | 41.43506944 |  |  |
| S116 | 109.8888917 | 41.43641389 |  |  |
| S117 | 109.8845028 | 41.43634167 |  |  |
| S118 | 109.8804056 | 41.43268889 |  |  |
| S119 | 109.8806417 | 41.42882778 |  |  |
| S120 | 109.8167667 | 41.38501111 |  |  |
| S121 | 109.786125 | 41.38119722 |  |  |
| S122 | 109.6912278 | 41.42454722 |  |  |
| S123 | 109.6861528 | 41.43234167 |  |  |
| S124 | 109.6856611 | 41.43417778 |  |  |
| S125 | 109.6815306 | 41.43950833 |  |  |
| S126 | 109.6796889 | 41.44878889 |  |  |
| S127 | 109.6831 | 41.45297222 |  |  |
| S128 | 109.6905694 | 41.451925 |  |  |
| S129 | 109.6886806 | 41.455175 |  |  |
| S130 | 109.6749806 | 41.45011667 |  |  |
| S131 | 109.6757639 | 41.45211667 |  |  |
| S132 | 109.6722222 | 41.44910278 |  |  |
| S133 | 109.6718139 | 41.45519444 |  |  |
| S134 | 109.6701528 | 41.45867222 |  |  |
| S135 | 109.6864694 | 41.47866667 |  |  |
| S136 | 109.6894306 | 41.47697778 |  |  |
| S137 | 109.6721694 | 41.47114167 |  |  |
| S138 | 109.8114333 | 41.38825556 |  |  |
| S139 | 109.8093361 | 41.38758889 |  |  |
| S140 | 109.8063278 | 41.39149444 |  |  |
| S141 | 109.8098139 | 41.39419444 |  |  |
| S142 | 109.8109889 | 41.395 |  |  |
| S143 | 109.8147444 | 41.39694722 |  |  |
| S144 | 109.8339806 | 41.25071389 |  |  |
| S145 | 109.8314639 | 41.26376389 |  |  |
| S146 | 109.8307778 | 41.270125 |  |  |
| S147 | 109.8304417 | 41.27010278 |  |  |
| S148 | 109.8270083 | 41.26010278 |  |  |
| S149 | 109.8294694 | 41.25493333 |  |  |
| S150 | 109.8330583 | 41.25058889 |  |  |
| S151 | 109.8344111 | 41.24283611 |  |  |
| S152 | 109.8016333 | 41.23282222 |  |  |
| S153 | 109.6805472 | 41.43889722 |  |  |
| S154 | 109.6785417 | 41.44731667 |  |  |
| S155 | 109.6801944 | 41.448725 |  |  |
| S156 | 109.68265 | 41.451925 |  |  |
| S157 | 109.6902472 | 41.45179722 |  |  |
| S158 | 109.6881 | 41.45890556 |  |  |
| S159 | 109.67495 | 41.45275 |  |  |
| S160 | 109.6715778 | 41.44883889 |  |  |
| S161 | 109.6711694 | 41.45538333 |  |  |
| S162 | 109.6844861 | 41.47902778 |  |  |
| S163 | 109.6896889 | 41.47762222 |  |  |
| S164 | 109.8089083 | 41.38740278 |  |  |
| S165 | 109.8080056 | 41.39155556 |  |  |
| S166 | 109.8107944 | 41.39483889 |  |  |
| S167 | 109.8140778 | 41.39741389 |  |  |
| S168 | 109.8177056 | 41.39744722 |  |  |
| S169 | 109.8223389 | 41.39229722 |  |  |
| S170 | 109.8385194 | 41.25161389 |  |  |
| S171 | 109.8359861 | 41.25516111 |  |  |
| S172 | 109.9690861 | 41.23458333 |  |  |
| S173 | 109.9734472 | 41.24556944 |  |  |
| S174 | 109.9663667 | 41.24570556 |  |  |
| S175 | 109.9625333 | 41.22811389 |  |  |
| S176 | 109.9610778 | 41.22374722 |  |  |
| S177 | 109.95965 | 41.22339722 |  |  |
| S178 | 110.0646389 | 41.27960556 |  |  |
| S179 | 110.0682083 | 41.27797778 |  |  |
| S180 | 110.0700111 | 41.28280556 |  |  |
| S181 | 110.0714167 | 41.28578611 |  |  |
| S182 | 110.0719694 | 41.286575 |  |  |
| S183 | 110.0635944 | 41.28690556 |  |  |
| S184 | 110.0638194 | 41.28669167 |  |  |
| S185 | 110.0546917 | 41.28526111 |  |  |
| S186 | 110.0547861 | 41.28500556 |  |  |
| S187 | 110.10725 | 41.28317222 |  |  |
| S188 | 110.1048333 | 41.27998889 |  |  |
| S189 | 110.0972222 | 41.27288056 |  |  |
| S190 | 110.1884722 | 41.28883889 |  |  |
| S191 | 110.1997 | 41.28346111 |  |  |
| S192 | 110.3190556 | 41.29176944 |  |  |
| S193 | 110.3504361 | 41.262025 |  |  |
| S194 | 110.3482472 | 41.26417222 |  |  |
| S195 | 110.3446639 | 41.27088056 |  |  |
| S196 | 110.3434 | 41.27308889 |  |  |
| S197 | 110.3605444 | 41.21160556 |  |  |
| S198 | 110.3602861 | 41.21174722 |  |  |
| S199 | 110.3596889 | 41.21431111 |  |  |
| S200 | 110.2935278 | 41.13477778 |  |  |
| S201 | 110.3064389 | 41.1207 |  |  |
| S202 | 110.3097306 | 41.11816944 |  |  |
| S203 | 110.3072278 | 41.11688889 |  |  |
| S204 | 110.305625 | 41.11621667 |  |  |
| S205 | 110.3283556 | 41.11086111 |  |  |
| S206 | 110.3112444 | 41.10668889 |  |  |
| S207 | 110.1171944 | 41.07781389 |  |  |
| S208 | 110.1166167 | 41.07784167 |  |  |
| S209 | 109.9731278 | 41.24553611 |  |  |
| S210 | 109.9599639 | 41.22352222 |  |  |
| S211 | 110.0647167 | 41.28309722 |  |  |
| S212 | 110.0706361 | 41.28180556 |  |  |
| S213 | 110.0685278 | 41.27835556 |  |  |
| S214 | 110.0766778 | 41.28457222 |  |  |
| S215 | 110.0779 | 41.28772222 |  |  |
| S216 | 110.0635778 | 41.28649444 |  |  |
| S217 | 110.1053778 | 41.283225 |  |  |
| S218 | 110.1158667 | 41.28205556 |  |  |
| S219 | 110.1908417 | 41.2864 |  |  |
| S220 | 110.1829083 | 41.28948611 |  |  |
| S221 | 110.1805556 | 41.29426944 |  |  |
| S222 | 110.175075 | 41.29828889 |  |  |
| S223 | 110.179075 | 41.29941944 |  |  |
| S224 | 110.1794694 | 41.29958889 |  |  |
| S225 | 110.1979167 | 41.28877778 |  |  |
| S226 | 110.2022556 | 41.28430278 |  |  |
| S227 | 110.2001083 | 41.28306111 |  |  |
| S228 | 110.319225 | 41.29119444 |  |  |
| S229 | 110.3501528 | 41.26189722 |  |  |
| S230 | 110.3486556 | 41.26374167 |  |  |
| S231 | 110.3486556 | 41.26488056 |  |  |
| S232 | 110.3505111 | 41.27079167 |  |  |
| S233 | 110.3580222 | 41.21208056 |  |  |
| S234 | 110.3605194 | 41.21062778 |  |  |
| S235 | 110.2903194 | 41.16179722 |  |  |
| S236 | 110.2902806 | 41.13778056 |  |  |
| S237 | 110.2921611 | 41.13706944 |  |  |
| S238 | 110.3111417 | 41.11701389 |  |  |
| S239 | 110.3298694 | 41.11085278 |  |  |
| S240 | 110.3431972 | 41.10227222 |  |  |
| S241 | 110.33435 | 41.11111111 |  |  |
| S242 | 110.3218389 | 41.11176667 |  |  |
| S243 | 110.3132833 | 41.11265278 |  |  |
| S244 | 110.1417917 | 41.07823333 |  |  |

Note: “S” stands for sampling point of *Astragalus mongholicus* Bunge
